# Selenium Speciation in the Fountain Creek Watershed (Colorado, USA) Correlates with Water Hardness, Ca and Mg Levels

**DOI:** 10.3390/molecules22050708

**Published:** 2017-04-30

**Authors:** James S. Carsella, Irma Sánchez-Lombardo, Sandra J. Bonetti, Debbie C. Crans

**Affiliations:** 1 Department of Chemistry, Colorado State University–Pueblo, 2200 Bonforte Blvd, Pueblo, CO 81001, USA; jim.carsella@csupueblo.edu (J.S.C.), sandra.bonetti@csupueblo.edu (S.J.B.); 2 Cell and Molecular Biology Program, Colorado State University, Fort Collins, CO 80523, USA; 3 Department of Chemistry, Colorado State University, Fort Collins, CO 80523, USA; 4 División de Ciencias Básicas, Universidad Juárez Autónoma de Tabasco, Cunduacán, Tabasco 86690, Mexico; irmas_l@yahoo.com.mx

**Keywords:** speciation, selenium determination, acidity, toxicity, thermodynamics

## Abstract

The environmental levels of selenium (Se) are regulated and strictly enforced by the Environmental Protection Agency (EPA) because of the toxicity that Se can exert at high levels. However, speciation plays an important role in the overall toxicity of Se, and only when speciation analysis has been conducted will a detailed understanding of the system be possible. In the following, we carried out the speciation analysis of the creek waters in three of the main tributaries—Upper Fountain Creek, Monument Creek and Lower Fountain Creek—located in the Fountain Creek Watershed (Colorado, USA). There are statistically significant differences between the Se, Ca and Mg, levels in each of the tributaries and seasonal swings in Se, Ca and Mg levels have been observed. There are also statistically significant differences between the Se levels when grouped by Pierre Shale type. These factors are considered when determining the forms of Se present and analyzing their chemistry using the reported thermodynamic relationships considering Ca^2+^, Mg^2+^, SeO_4_^2−^, SeO_3_^2−^ and carbonates. This analysis demonstrated that the correlation between Se and water hardness can be explained in terms of formation of soluble CaSeO_4_. The speciation analysis demonstrated that for the Fountain Creek waters, the Ca^2+^ ion may be mainly responsible for the observed correlation with the Se level. Considering that the Mg^2+^ level is also correlating linearly with the Se levels it is important to recognize that without Mg^2+^ the Ca^2+^ would be significantly reduced. The major role of Mg^2+^ is thus to raise the Ca^2+^ levels despite the equilibria with carbonate and other anions that would otherwise decrease Ca^2+^ levels.

## 1. Introduction

The environmental levels of Se are regulated and strictly enforced in the USA by the Environmental Protection Agency (EPA) [1,2,3,4] because even though low levels are beneficial high levels are toxic [5,6,7,8,9,10]. The high Se toxicity on aquatic life and particularly fish reproduction [11,12,13,14] has led to the development of a chronic exposure criterion [11]. High Se levels are often found when waterways run over the Se-rich shale deposits [3] or when agricultural runoff carries Se into waterways from Se rich soils [15,16,17,18]. Specifically, the Lower Fountain Creek (LF) in southeastern Colorado is a known and major contributor to the Se levels downstream from its confluence with the Arkansas River (AR); this confluence is in the eastern part of the city of Pueblo, Colorado (for a map see [3,19]). These high Se levels in the Fountain Creek and in the Arkansas River increase the potential of damaging environmental effects downstream in the Arkansas River basin [3]. While Se toxicity is linked to particular species [5,12,13,14,16,20] and the presence of selenite (SeO_3_^2−^) [21,22], many environmental studies only measure and report the total amount of elemental Se. When analyzing and investigating Se speciation, a better understanding of the interaction of Se species in a natural environment is gained [5,7,21,23,24,25,26,27,28]. Recent studies of fish in the Fountain Creek Watershed demonstrated that high numbers of fish species can be observed at sites with high levels of Se [21]. This work led to the hypothesis that the toxicity is dependent on the presence of toxic Se species and not the total Se level [21] and support the need for additional studies that consider the effects of Se species on different types of fish. The following manuscript analyzes the chemistry of Se speciation in these waters and that of two major cations, Ca^2+^ and Mg^2+^, using the thermodynamic parameters.

Total Se concentrations in aqueous environments generally vary between 0.06 to 400 µg/L [29,30,31,32]. The most common forms of Se in surface waters are Se(IV) and Se(VI) with some reports also including Se(II) in either organic or inorganic form [8,15,33,34,35]. Selenium is most soluble in aqueous solution under oxidizing conditions and the distribution of the oxidized forms of Se, selenite (SeO_3_^2−^) and selenate (SeO_4_^2−^) depend on specific conditions [8,15,34,35]. The less soluble and reduced form of Se, Se^2−^, exists complexed in the earth’s crust, and may also be found at low levels in reducing water environments [8]. Pourbaix diagrams are important to describe the speciation of Se in idealized systems [8,15,21,34,35]. However, considering that the Pourbaix diagrams are calculated assuming specific conditions, for example, where the concentration and ionic strength do not affect the speciation; such representations are at best, a first approximation [15,21,34,35]. From the reported Pourbaix diagrams, it is clear that selenous acid (H_2_SeO_3_) is favored at the conditions normally found in oxidizing natural stream waters (pE ≈ 13.5) at a pH < 3.0 [36]. Hydrogen selenite (HSeO_3−_) is favored in the same oxidizing conditions up to about pH 5.0. Above pH 5.0 selenate (SeO_4_^2−^) is the most favored Se species. Using this information, it follows that in less aerated waters (pE ≈ 13.5) at pH values between 3 and 8, hydrogen selenite, HSeO_3_^−^ is the favored species [21,34]. However, recently we demonstrated that for Fountain Creek the Se was about 90% Se(VI) and 10% Se(IV) even through the Pourbaix diagrams predicted more Se(IV) [21]. In the following, we explore the following hypothesis: *classical speciation plots are effective in describing the Se speciation when considering two influential cations, Ca^2+^ and Mg^2+^*. The conversions between the different oxidation states of Se are reported to be slow [35] and it is possible that the speciation in the watershed does not represent equilibrium conditions. Previously a correlation between total elemental Se with water hardness [19] was reported. However, the detailed chemistry of this correlation involving a combination of Ca^2+^ and Mg^2+^ was not investigated [19]. Specifically, the possibility of the formation of the soluble and insoluble forms of CaSeO_4_ correlate with the observed aqueous Se levels and equilibrium conditions.

In the following, the Se speciation chemistry of waters in the Fountain Creek Watershed sites was explored. Since the two most common cations determining water hardness are calcium (Ca^2+^) and magnesium (Mg^2+^), in addition to Se, Ca, and Mg levels were measured at the Fountain Creek sites. Furthermore, the speciation chemistry and its relationship to Se content with Ca^2+^ and Mg^2+^ contents were explored. Samples were collected from three different Fountain Creek reaches: Upper Fountain Creek (UF), Monument Creek (MC) and the previously mentioned LF portion of Fountain Creek. The Se concentrations, the pH, and the concentrations of Ca^2+^ and Mg^2+^ were determined. Since correlations have been reported between Se levels and water hardness, the current manuscript explores the chemical speciation of Se, Ca, and Mg. The goal was to examine if specific chemical relationships between Se, Ca^2+^, and Mg^2+^ combined can explain the reported correlation with water hardness. Speciation diagrams show that the Se content indeed correlates with the soluble CaSeO_4_ levels even though the thermodynamic equilibria of Se speciation alone are not sufficient to describe all the properties of this system.

## 2. Results 

### 2.1. Characterization of the Se-Levels and Speciation in the Fountain Creek

**Figure 1:**
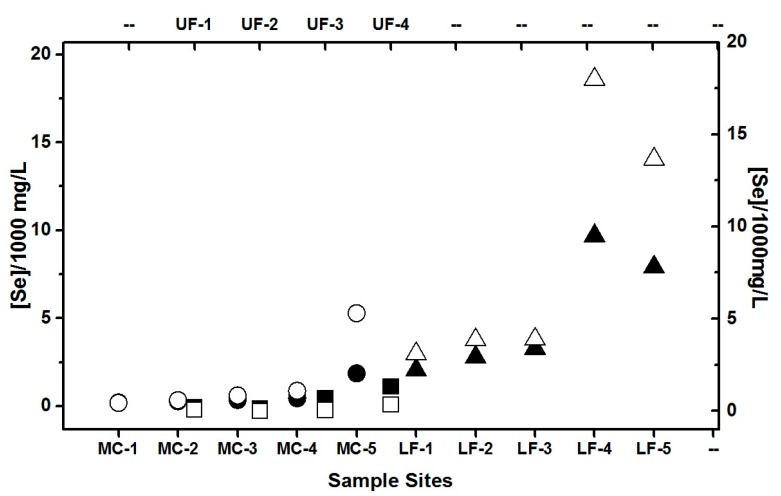
Total Se concentrations in the creek water (solid symbol) spring and (open symbol) fall 2007 in µg/L. UF stands for Upper Fountain Creek (squares), MC Monument Creek (circles) and LW, Lower Fountain Creek (triangles) [21]. Data points were measured in triplicates and details of the sampling site was described.

Fountain and Monument Creeks were sampled for dissolved Se in the past [3,4,18,19,21,37,44,45,46]. High levels of Se were reported (3.4–12 µg/L) [3] with up to 64 µg/L [3] between Colorado Springs and Pueblo, Colorado. Selenium originates from the Pierre Shale located in southeastern Colorado [2] which is underlying both Fountain and Monument Creeks. Fourteen sites were monitored in the spring and fall of 2007 (Table 1, Figure 1) and part of this data was reported previously [21,37]. Se concentrations and cation concentrations were measured using inductively coupled plasma–mass spectrometry (ICP–MS) and the results with Ca^2+^ and Mg^2+^ are now reported in this manuscript. In Table 1 the Se, pH, and alkalinity reported previously are listed for all water samples. The Ca and Mg concentrations and temperature are reported here. As previously shown, the temperature and pH influence speciation of different elements and these parameters were investigated here.

Several Analyses of Variances (ANOVA) tests were conducted on the Se levels from all reaches to determine which Se measurements are statistically different between site, reach, shale type and Ca, and Mg levels. In Figure 1, the observed Se concentrations are plotted at each site for both spring and fall.

Due to the exponential increase in the Se, Ca, and Mg concentrations in the reaches of the sampled creeks, natural log transformations were used to fit the data to a normal distribution to carry out the ANOVA analysis. An ANOVA test was also conducted on the Se, Ca, and Mg levels from the Pierre shales (Pierre Shale (PS), Upper Pierre Shale (UPS), Lower Pierre Shale (LPS), Continuous Pierre Shale (CPS) and No Pierre Shale (NPS)). A statistically significant difference in Se levels exists between LF and both the UF and MC reaches (*p* < 0.0005). No statistically significant difference exists between the UF and MC reaches of the Fountain Creek Study area. The ANOVA with Tukey’s pairwise comparisons results also show a statistically significant difference between the Se levels and the Shale type (*p* < 0.0005). The results indicate that a statistically significant difference exists between the Se levels in the water exposed to LPS and the rest of the PS formations as well as NPS area of the study area. In addition, there is a statistically significant difference between the Se levels in the water in the UPS and the area of NPS. No statistically significant differences were found between the NPS and CPS. A statistically significant difference exists between all reaches for Ca and Mg levels (*p* < 0.0005) with the lowest levels in MC. The ANOVA between shale type and Mg indicates that a statistically significant difference exists between LPS and UPS and NPS. There is an overlap between CPS and all shale types with respect to Mg levels. The Ca levels are statistically significantly different in all shale types except for the CPS in the spring data set. In the fall Ca dataset, there is an overlap between the levels in the CPS and NPS as well as LPS and UPS. A statistically significant difference exists between CPS-NPS and UPS-LPS (*p* < 0.0005).

**Figure 2:**
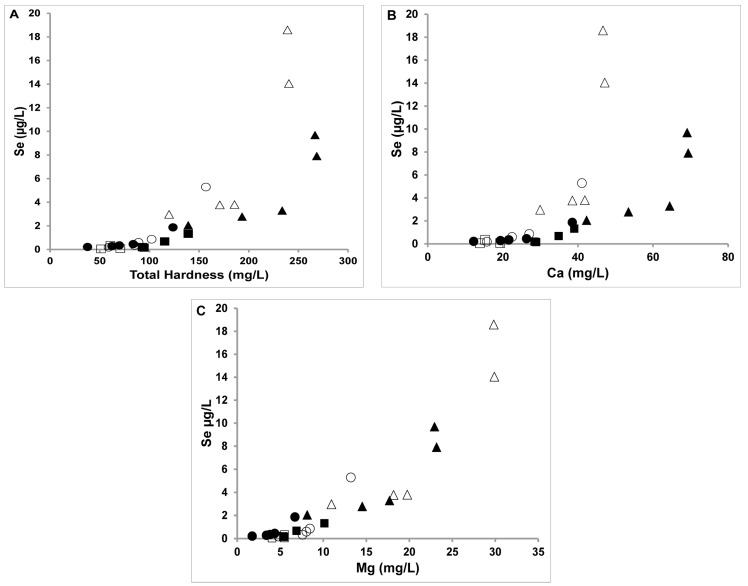
Total Se level ([Se]/µg/L) vs Total Hardness (**A**), Total Ca level (**B**) and Total Mg level (**C**) in the creek water (solid symbol) spring and (open symbol) fall 2007 in µg/L. UF is the Upper Fountain Creek (squares), MC Monument Creek (circles) and LF Lower Fountain Creek (triangles) [21]. Data points were measured in triplicates and details of the sampling sites were described.

The highest Se concentrations were observed at the MC-5 site that is in Colorado Springs and at the LF-4 and LF-5 sites. The latter sites are in the city of Pueblo (during both, spring and fall), see Figure 2, and for a map see [3,19]. These sites with very high Se levels are located where the human use is becoming more prevalent and thus include both contributions from natural and human waste sources [21,37]. The highest Se concentration in Monument Creek at MC-5 could be attributed to the presence of UPS under Monument Creek, while the elevated Se concentrations in the LF-4 and LF-5 sites are due to the LPS formation under the creek [19].

### 2.2. Se, Ca and Mg Levels and Hardness in the Fountain Creek Water

Because correlations were reported between Se levels and water hardness by Herrmann and coworkers [19], the current manuscript explores the chemical speciation of Se, Ca and Mg to examine if specific chemical relationships between Se and Ca or Mg can explain the reported correlation with water hardness. In Figure 2, the Se concentrations are plotted as a function of total hardness, and a correlation was suggested for the final sites at the Fountain Creek Watershed. Because water hardness is generally attributed to the amount of calcium (Ca^2+^) and magnesium ions (Mg^2+^) dissolved in the water, we investigated the relationships by plotting Se level as a function of the hardness (in Figure 2A), the concentrations of Ca^2+^ (Figure 2B) and the concentration of Mg^2+^ (Figure 2C). The plots show that more Ca was measured in the water in spring as compared to the fall and that this difference correlates with the difference observed in the hardness.

**Figure 3:**
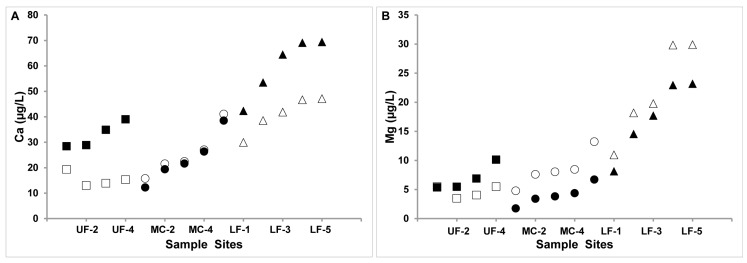
(**A**) Total calcium concentrations (presumed Ca^2+^) in the creek water (solid symbols) spring and (open symbols) fall year in mg/L and (**B**) total magnesium concentration (presumed Mg^2+^) in the creek water (solid symbols) spring and (open symbols) fall year in µg/L. UF is the Upper Fountain Creek (symbol squares), MC Monument Creek (symbol circles) and LF Lower Fountain Creek (symbol triangles). Data points were measured in triplicates and details of the sampling sites were described [21].

Based on Figure 2 plots of Se versus Ca^2+^ and Se versus Mg^2+^ as well as plots of each cation versus collection site shown in Figure 3, the Se levels increase as the creek runs downstream and across the PS formation. Variations in Se levels exist for each cation and water sample location and some different patterns emerge between the three parameters. Specifically, in the fall the Se concentrations are higher than in the spring for most samples. Linear relationships can be suggested between Se levels and hardness, and between Se and Mg^2+^ levels for both fall and spring. Linearity is also observed for Se levels and Ca^2+^ levels measured in the spring, but not in the fall. This change in the pattern is of interest because it could be related to uptake of Se by plants in the creek waters, and this will be the topic of a future investigation factoring in measurements of Se(IV) and Se(IV) [19]. Most UF and MC sites that have not been exposed to the PS formation, exhibit characteristically low Se concentrations in the water. The Se levels begin to rise at the UF-4 and MC-5 sites which are at the intersections of the UF reach with the CPS formation and the MC reach with the UPS formation. The water from both the UF and MC reaches confluence with the LF reach and flow into the LF-1 site. The most interesting pattern shown here is the correlation of the Se levels with Ca^2+^ concentrations in the spring measurements and with the Mg^2+^ concentrations in both spring and fall seasons.

In the case of the Mg^2+^, there is less seasonal variation (Figure 2C). In Figure 3B the concentrations of Mg at the different sample sites were plotted, and the same trend for the sample locations was found for the Mg levels in samples collected in the fall or spring (see Figure 2 and Figure 3). The UF-4, MC-5, LF-4 and LF-5 sites contain the highest levels of Ca in spring. Therefore, the fact that the Se levels correlate differently with the hardness in the fall versus the spring streams can be attributed to the seasonal variations in Ca^2+^ levels shown in Figure 2B. We suggest that although Mg^2+^ is important for defining the environment in these systems because the concentrations of Mg^2+^ remain constant, it influences hardness by facilitating dissolution of insoluble Ca^2+^ species as has been reported previously for MgCO_3_-CO_2_ systems [47,48]. We suggest that similar effects exist for the MgSeO_3_-SeO_2_ system and that this can explain at least in part the concentrations of SeO_3_^2−^ and SeO_4_^2−^ systems above those expected by the solubility products.

### 2.3. Speciation Profile for Sites with Low and High Levels of Se

**Figure 4:**
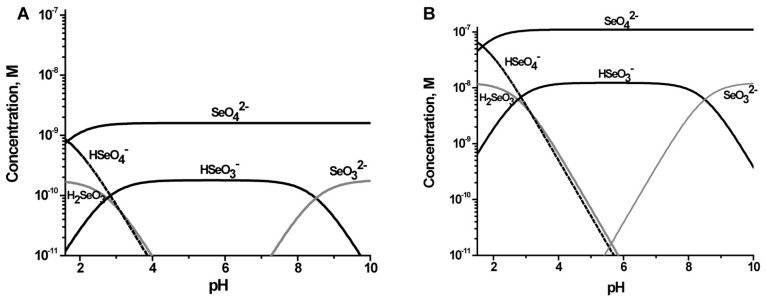
Speciation diagrams were calculated for two different sites along the Fountain Creek in the stream water: (**A**) The low concentration UF-2 sites with [Se]_tot_ 3.36 × 10^−10^ M, and (**B**) LF-4 with [Se]_tot_ 1.22 × 10^−7^ M. Recently the speciation was determined to around 90% Se(VI) for several of the sites studied in this work [25].

In Figure 4 we show the distribution of Se species that would be anticipated if the most of the oxidized Se species present are in a thermodynamic equilibrium using the reported formation constants (see the Materials and Methods section for formation constants). The figures show that under oxidizing conditions the major species is predicted to be SeO_4_^2−^ from pH 5 to 8, but that at higher concentrations the contribution of Se(IV) to Se(VI) is smaller. However, it must be noted, that these calculations are done at oxidizing conditions, and as reported previously using Pourbaix diagrams, when the pE measured at these sites along the Fountain Creek is used, the predicted major Se component was Se(IV) [21]. However, speciation studies showed that regardless, about 90% of the Se was found to be in oxidation state VI [21] and using these results we calculated the speciation that would be observed for the UF-2 and LF-4 sites (Figure 4).

**Figure 5:**
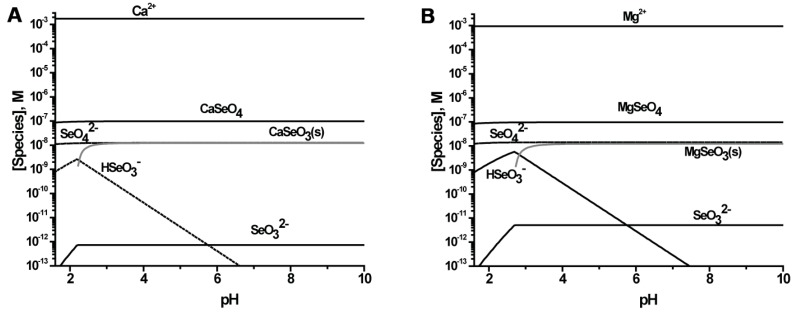
Speciation diagrams were calculated for LF-4 site along the Fountain Creek in the stream water with concentrations (**A**) of [Se(IV)] 1.2 × 10^−8^ M, [Se(VI)] 1.1 × 10^−7^ M and [Ca]_tot_ 1.7 × 10^−3^ M and (**B**) [Se(IV)] 1.2 × 10^−8^ M, [Se(VI)] 1.1 × 10^−7^ M and [Mg]_tot_ 9.4 × 10^−4^ M.

Ca^2+^ is known to form both soluble and insoluble Ca^2+^–SeO_4_^2−^ species and the distribution of these species was investigated using the LF-4 site concentrations of Se (Figure 5A). The levels of the soluble form of CaSeO_4_ was determined previously in other waters in studies using isotopically pure materials. These studies confirmed the presence of a significant amount of oxidized Se(VI) in such waters [47,48].

Studies with the Fountain Creek waters have previously shown that the formation of CaSeO_4_ is competing with the formation of Ca^2+^ complexes with CaSeO_3_, CO_3_^2−^, and with HCO_3_^−^; and the latter will be discussed below. Figure 5A shows the speciation of Se(IV) and Se(VI) in the presence of Ca^2+^ showing more CaSeO_4_ than CaSeO_3_. As in the case of the Ca^2+^, the Mg^2+^ speciation was considered in Figure 5. The speciation diagram was constructed for the Mg and Se species using the LF-4 site concentrations, showing that the most favorable species in the water under these conditions were MgSeO_4_ and the insoluble MgSeO_3_.

**Figure 6:**
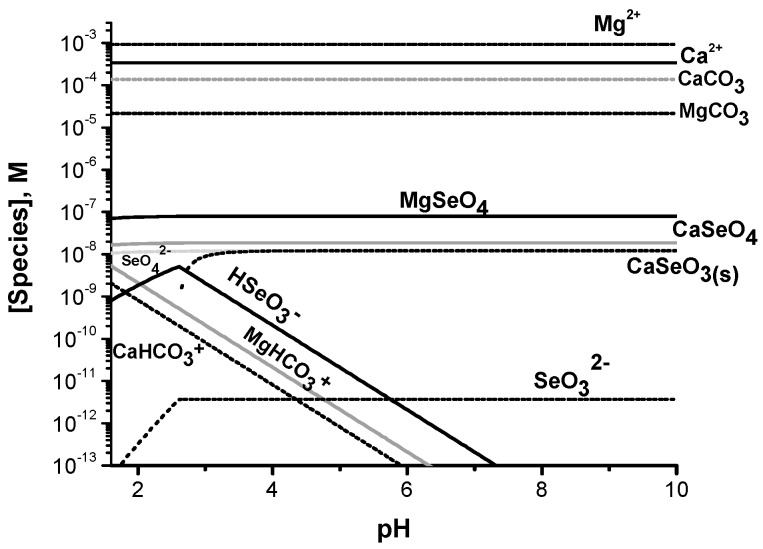
Speciation diagram was calculated for the LF-4 site along the Fountain Creek in the stream water with concentrations of [Se(IV)] 1.2 × 10^−8^ M, [Se(VI)] 1.1 × 10^−7^ M, [Ca]_tot_ 1.7 × 10^−3^ M and [Mg]_tot_ 9.4 × 10^−4^ M.

As elevated concentrations of Ca^2+^ and Mg^2+^ were measured, the speciation diagrams for both cations were plotted in Figure 5A and by comparison, we include the speciation diagram for the Se, HCO_3_^−^ and CO_3_^2−^ species with both Ca^2+^ and Mg^2+^ together in Figure 6. As expected, the carbonate species with both cations are the most abundant since their equilibrium constants are the highest.

The carbonates thus shift the selenite equilibrium for the Mg^2+^ since no insoluble MgSeO_3_ is present; however, this is not the case for the CaSeO_3_ that continues to have insoluble material. The presence of carbonates with their large equilibrium constants and the higher concentration of Ca^2+^ versus Mg^2+^ would support the formation of insoluble calcium selenite. This behavior could be a consequence of a cycle whereby carbonates are consumed by organisms present in the water leading to more release of Ca^2+^. This is a logical explanation given that the pH of the Fountain Creek water (Table 1) is slightly alkaline and the alkalinity measurements indicate that under these conditions the waters are oversaturated with Ca^2+^ according to the Langelier saturation index (LSI) [49] The LSI was calculated for the LF-4 site in the summer using the alkalinity value of 140 mg/L of CaCO_3_ and the K_sp_ for CaCO_3_ (6.92 × 10^−9^) where LSI = log([Ca^2+^][CO_3_^2−^]/K_sp_) = 2.45; this indicates that the water is saturated with CaCO_3_ which would allow precipitation of some CaCO_3_ particles in the water.

Although the formation constant for CaCO_3_ is large, at pH values near neutral the concentration of CO_3_^2−^ in the creek waters is very low. The protonated form of HCO_3_^−^ is present, but the complex that it forms with Ca^2+^ is much less stable. The methods used in this study specifically preclude measurements of carbonate and the values are thus not routinely measured. However, carbonate levels in river waters have been reported previously and are in the range of 20–200 mg/L CaCO_3_ [49,50]. To illustrate the levels of calcium carbonates in these systems, we assume a carbonate level of 140 mg/L in the speciation diagram calculated in Figure 6. Since the formation constant for the soluble CaSeO_4_ is high (K = 10^2.68^) a significant portion of the soluble Se presumably exists as a soluble form of CaSeO_4_, however, the solubility product for the insoluble CaSeO_4_ may be reached at some of the sites with high Se concentrations. The possibility that the CaSeO_4_ species is a major species involved in the correlation between Se levels and hardness was considered, and the speciation diagrams including the Ca^2+^ cations were examined at the LF-4 site concentrations and are shown in Figure 6. As seen from Figure 6 the major species in solution are indeed CaSeO_4_ and Ca(HCO_3_)_2_.

At the pH values (5 < pH < 8) of the water that has been investigated, most of the carbonate would be protonated and not as prone to precipitation. In addition, Castanier and coworkers show that production of carbonate particles by heterotrophic bacteria follows different paths and these induce a pH increase and an accumulation of CO_3_^2−^ and HCO_3_^−^ ions in the system [51]. This is important because the high concentration of Ca^2+^ found in the water correlates with the total Se and because in our previous work [21] we found that the major Se species is present in the nontoxic form of Se(VI) as SeO_4_^2−^. However, additional analysis is needed and will be the topic of future communications. The subject of other cations will be considered particularly when the processing of the Se due to uptake by the plants in the creek bed is examined. Thus, speciation of iron and other metal ions become important [25,52,53,54,55,56,57].

### 2.4. Exploration of Other Physical Parameters and Their Impact on the Speciation

To further explore the effects of other physical parameters the Se, Ca and Mg levels were measured in addition to pH, temperature, and discharge. In this study fourteen sites were monitored in spring and fall of 2007 (Table 1). We found that the highest Se concentrations in each reach were observed at the UF-4 and MC-5 sites in Colorado Springs and LF-4 and LF-5 sites in Pueblo in both seasons as shown in Figure 2.

**Figure 7:**
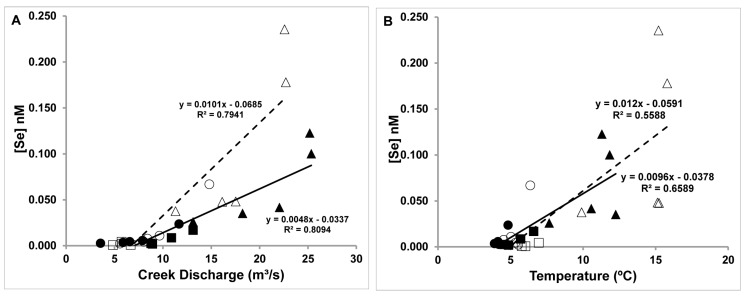
Total Se concentration in nM vs. Creek discharge in m^3^/s (**A**) and Se concentration in nM vs. water temperature (°C) (**B**). Selenium concentrations in the creek water (solid symbol) spring and (open symbol) fall 2007 in nM. UF stands for Upper Fountain Creek (squares), MC Monument Creek (circles) and LW, Lower Fountain Creek (triangles) Regression lines are shown as solid for spring and dashed for fall. Details of the sampling sites were described previously [21].

The water temperature values are more consistent within a season (Table 1) and the water temperatures are colder during the spring. The discharge rates are higher during the spring runoff and the lower discharge during the fall resulted in higher Se concentrations (Figure 7A). This is consistent with the observation of higher Se levels when there are higher water temperatures and smaller discharge rates; this allows for more dissolution of Se into the creek water. The total Se content in the water during spring and fall correlates with the water temperature and the creek discharge rate as shown from the plots in Figure 7.

**Figure 8:**
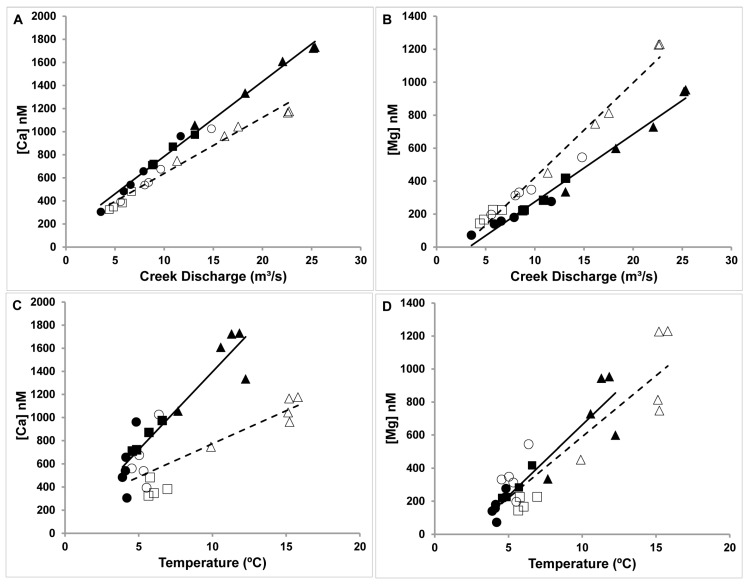
Total Ca concentration in nM plotted vs. creek discharge in m^3^/s (**A**). For comparison, total Mg concentration in nM plotted vs. creek discharge in m^3^/s (**B**). Total Ca concentration in nM plotted vs. water temperature (°C) (**C**). Total Mg concentration in nM plotted vs. water temperature (°C) (**D**). concentrations in the creek water (solid symbol) spring and (open symbol) fall 2007 in µg/L. UF stands for Upper Fountain Creek (squares), MC Monument Creek (circles) and LW, Lower Fountain Creek (triangles) Regression lines are shown as solid for spring and dashed for fall. Details of the sampling sites were described previously [21].

The best relationship to dissolved Se in the creek exists with flow rate (Figure 7A, R^2^_spring_ = 0.8094, R^2^_Fall_ = 0.7941). Temperature is also correlated with dissolved Se but the correlation is not as strong as the flow rate (Figure 7B). Together the data for temperature and flow rate suggest that there are symbiotic effects on dissolved Se and that these parameters assist in increasing the amount of dissolved Se, and because these parameters affect the Ca^2+^ (Figure 8A,C) and Mg^2+^ (Figure 8B,D) in a similar manner, they contribute to increasing the Ca^2+^ level above that expected for the solubility product (Figure 8).

## 3. Materials and Methods

### 3.1. Materials and Reagents

The standards for EPA method 200.8 were purchased as a NIST traceable multi-element custom made standard from Inorganic Ventures (IV, Christiansburg, VA, USA, CSTU-STD-1) and from SPEX CertiPrep (Metuchen, NJ, USA, part# CL-CAL-2A). The IV standard was made to contain 1000 µg/mL of Ca, Mg, Na, Fe, K. Furthermore, it contains 10 µg/mL Al, Sb, As, Ba, Cd, Se, Ag, Pb, Be, Co, Cr, Zn, Cu, Mn, Mo, Ni, V, U, Th, Tl. The SPEX standard was diluted to 50 ppb and used as a check standard to verify calibration. The internal standards used were (Inorganic Ventures 2008ISS-125, ^6^Li, Sc, Y, In, Tb, Bi, Ho) and Germanium (Ge) (Inorganic Ventures MSGE-10PPM-125ML). The nitric acid (A467-500, Fisher Scientific, Waltham, MA, USA) and hydrochloric acid (Fisher A466-500) used in these procedures were purchased as Optima grade from Fisher Scientific.

### 3.2. Fountain Creek Sampling

Water quality measurements of surface waters included measuring temperature, pH, specific conductance, and dissolved oxygen per the protocols described by the United States Geological Survey (USGS) protocols [19,58]. Details of the sampling site was described previously including the Upper Fountain creek, the Monument Creek and the Lower Mountain Creek [21]. Each parameter was measured three times at each site over a 10-day period. The measurements were made at 0, 5 and 10-day intervals. Water samples were taken at equivalent distance points on a line across the creek at each sampling site. The samples were collected in 250 mL low density polyethylene (LDPE) plastic bottles and stored on ice for transport. The collection was performed per limnologic sampling protocol by holding the bottle with the mouth of the container facing downstream at an angle near 45°. The container was rinsed with creek water 3 times prior to taking the sample. Triplicate samples were collected at each site during each sampling interval and filtered through a 0.45 micron reconstituted cellulose membrane syringe filter (Phenex AF0-8103-12) purchased from Phenomenex (Torrance, CA, USA) [58]. After filtering, water samples including field blanks for each trip were preserved with 1% Optima grade HNO_3_. The sample was divided in two, one was analyzed and the other stored at 4 °C. Six milliliters were used for cation determination by ICP–MS.

### 3.3. Analysis for Se, Ca and Mg

Water samples were analyzed for Se, Ca and Mg on a 7500ce ICP–MS (Agilent, Santa Clara, CA, USA) following the ICP-MS drinking water method, EPA Method 200.8 [59]. Since these samples were not intended for evaluation for municipal drinking water, the ICP–MS was run with the Octopole Reaction System (ORS). Hydrogen was used as a reaction gas for ^40^Ca and ^78^Se measurements. The elements ^24^Mg and ^44^Ca were measured using helium as a collision gas, to reduce interferences. Multi-element environmental external calibration standard (CSTU-STD-1) and internal standards were diluted in 1% nitric and 0.5% hydrochloric acid prior to analysis. The internal standard was diluted to a final concentration of 20 ppb from a 10-ppm stock. Germanium was added to the final internal standard mix before dilution to make a final concentration of 20 ppb. The Ge was used as an internal standard for Se because of Ge having a closer ionization potential to Se. Scandium was used as the internal standard for Ca and Mg.

### 3.4. Alkalinity

Alkalinity was measured by titration on all samples collected using the protocols listed in the book *Standard Methods for the Examination of Water and Wastewater* for alkalinity [60]. Details of each sampling site was described previously including the Upper Fountain creek, the Monument Creek and the Lower Mountain Creek [21].

### 3.5. Statistical Calculations

Analysis of Variance was used to determine if statistically significant differences exist between the water components (Ca, Mg, and Se) exposed to Pierre Shale type, creek reach, and sites. Creek discharge rates were obtained from USGS monitoring sites via web interfaces [19,58]. The sites nearest the monitoring stations (UF-1, UF-4, MC-1, MC-2, MC-4, LF-1, LF-2, and LF-4) used the data directly from the monitoring stations. The sites between the stations (UF-2, UF-3, MC-3, MC-5, LF-3, and LF-5) used a weighted average of the flow rates measured at the USGS stations above and below the sampling site used in this study.

### 3.6. Speciation Calculations

Species distribution diagrams were calculated by using HYSS 2003 software [61]. The concentration using for the consideration described here for the UF-2 site was [Se]_tot_ 3.36 × 10^−10^ M, and for LF-4 site was [Se]_tot_ 1.22 × 10^−7^ M. When the speciation diagrams with Ca and Mg cations were constructed the concentrations used were [Se(IV)] 1.2 × 10^−8^ M, [Se(VI)] 1.1 × 10^−7^ M, [Ca]_tot_ 1.7 × 10^−3^ M and [Mg]_tot_ 9.4 × 10^−4^ M. The label Cr designate crystalline phase, see below constants for CaSeO_3_·H_2_O_(cr)_ and MgSeO_3_·6H_2_O_(cr)_ at 25 °C and *I* = 0 [62]. The speciation diagrams were constructed using the following equilibrium reactions and constants [62]:

H_2_SeO_3_ ⇌ H^+^ + HSeO_3_^−^  K_2_ = 10^−2.75^

HSeO_3_^−^ ⇌ H^+^ + SeO_3_^2−^  K_1_ = 10^−8.5^

HSeO_4_^−^ ⇌ H^+^ + SeO_3_^2−^  K_1_ = 10^−1.66^

Ca^2+^ + H_2_O_(l)_ + SeO_3_^2−^ ⇌ CaSeO_3_·H_2_O_(cr)_  K = 10^6.4^

Ca^2+^ + 2H_2_O_(l)_ + SeO_4_^2−^ ⇌ CaSeO_4_·2H_2_O_(cr)_  K = 10^2.68^

Ca^2+^ + SeO_4_^2−^ ⇌ CaSeO_4(aq)_  K = 10^2.0^

Mg^2+^ + 6H_2_O_(l)_ + SeO_3_^2−^ ⇌ MgSeO_3_·6H_2_O_(cr)_  K = 10^5.82^

Mg^2+^ + 6H_2_O_(l)_ + SeO_4_^2−^ ⇌ MgSeO_4_·6H_2_O_(cr)_  K = 10^1.13^

Mg^2+^ + SeO_4_^2−^ ⇌ MgSeO_4(aq)_  K = 10^2.2^

Mg^2+^ + CO_3_^2−^ ⇌ MgCO_3(aq)_  K = 10^3.24^

Mg^2+^ + HCO_3_^−^ ⇌ MgHCO_3(aq)_  K = 10^1.23^

Ca^2+^ + CO_3_^2−^ ⇌ CaCO_3(aq)_  K = 10^4.48^

Ca^2+^ + HCO_3_^−^ ⇌ CaHCO_3(aq)_  K = 10^1.25^

CaCO_3_↓ ⇌ Ca^2+^ + CO_3_^2−^  K_sp_ = 4.55 × 10^−9^ [47,48]

## 4. Conclusions

A speciation analysis was carried out using Se and pH data on creek samples from 14 sites in the Fountain Creek Watershed which were supplemented with new measurements of Ca, Mg and temperature at these same sites. Details of the sampling sites were described previously including the Upper Fountain Creek, the Monument Creek and the Lower Mountain Creek locations [21]. Several ANOVA analyses were conducted on the Se levels from all reaches to consider which Se measurements are statistically different between site, reach, the type of shale and Ca, and Mg levels. An ANOVA was also performed on the Pierre shales (Pierre Shale (PS), Upper Pierre Shale (UPS), Lower Pierre Shale (LPS), Continuous Pierre Shale (CPS) and No Pierre Shale (NPS)) and a statistically significant difference in Se levels exists between LF and both the UF and MC reaches (*p* < 0.0005). The subsequent analysis supports the interpretation that the Ca^2+^ ion is mainly responsible for the observed high Se levels in these waters presumably by the formation of the soluble and insoluble CaSeO_4_. Although the Mg^2+^ levels also correlate with the Se level, this cation is not present at the same high level and thus presumably serves mainly to increase the Ca^2+^ levels beyond the solubility limit through ion-pair formation as reported previously [52]. These findings are important because several of these sites are found to have high levels of Se. However, previously it was demonstrated that there are no apparent toxicity effects associated with the fish population and these high Se levels [19,25,52,53,54,55,56,57]. The data presented here thus further characterize the Se-rich waters in which most of the Se is in the Se(VI) oxidation state. The studies here validate the observation that Se toxicity is reduced because Se(VI) is the predominant species and the studies presented here suggest that a major part of the Se(VI) is in the form of CaSeO_4_.

The detailed analysis of the Se species and its ability to explain observations underline the importance of consideration of the speciation chemistry in these environmental systems. The studies demonstrate that measurement of Se species allows for additional insights into the processes in a hydrological system. The detailed considerations including Mg^2+^ and Ca^2+^ allowed for an insight into the form of Se and the environmental system at hand. These considerations do require measurements of the oxidation state of the Se species and demonstrate the need for continued development of advanced methods for detection and measurements of components in complex matrices. We illustrate in this manuscript that the use of speciation analysis significantly enhances the understanding of previously reported results for other systems [25,52,53,54,55,56,57].

## Figures and Tables

**Table 1:** Se levels in the water along the Fountain Creek Watershed [21,37] ^a^.

Sample Sites	Spring									Fall								
	T (°C)	pH	Water Se (µg/L)	Std. Error Water Se	Water Ca (µg/L)	Std. Error Water Ca	Water Mg (µg/L)	Std. Error Water Mg	Alkalinity (mg CaCO_3_/L)	T (°C)	pH	Water Se (µg/L)	Std. Error Water Se	Water Ca (µg/L)	Std. Error Water Ca	Water Mg (µg/L)	Std. Error Water Mg	Alkalinity (mg CaCO_3_/L)
UF-1	4.6	7.2	0.193	0.003	28,440	892.5	5354	140.2	72	5.8	8.2	0.063	0.007	19,307	582.3	5481	261.7	76
UF-2	4.9	7.2	0.140	0.017	28,867	1993.3	5474	341.9	50	5.7	8.1	0.000	0.007	13,003	1355.7	3481	450.2	63
UF-3	5.7	7.6	0.673	0.090	34,863	2409.8	6890	456.9	81	6.0	8.1	0.043	0.009	13,897	291.7	4041	61.7	146
UF-4	6.6	7.6	1.323	0.313	39,017	2989.2	10,134	1504.9	83	7.0	8.1	0.340	0.010	15,303	205.1	5490	109.3	154
MC-1	4.2	7.4	0.207	0.012	12,227	357.3	1742	25.1	66	5.5	7.9	0.177	0.009	15,757	99.4	4772	102.3	86
MC-2	3.9	7.5	0.277	0.007	19,380	875.6	3400	253.9	74	5.3	8.0	0.330	0.023	21,547	394.7	7599	223.0	89
MC-3	4.1	7.2	0.343	0.017	21,623	1374.0	3818	283.5	73	4.5	8.1	0.597	0.038	22,450	283.6	8043	143.3	88
MC-4	4.1	7.5	0.443	0.023	26,327	1978.4	4365	324.2	78	5.0	8.0	0.860	0.021	27,047	91.3	8450	26.5	86
MC-5	4.8	7.4	1.863	0.216	38,503	3260.0	6712	538.8	110	6.4	8.1	5.283	0.269	41,080	172.1	13,217	416.0	105
LF-1	7.7	7.5	2.050	0.202	42,303	2751.5	8135	538.8	104	9.9	8.1	2.970	0.072	29,947	312.5	10,960	573.0	108
LF-2	12.2	7.2	2.780	0.008	53,465	698.1	14,540	714.4	141	15.2	8.1	3.773	0.060	38,537	520.7	18,177	137.8	179
LF-3	10.6	7.6	3.290	0.125	64,447	2316.7	17,703	514.4	138	15.1	8.1	3.800	0.057	41,837	806.5	19,770	182.5	180
LF-4	11.3	7.7	9.687	0.617	69,083	827.5	22,943	627.7	140	15.2	8.1	18.59	1.131	46,683	254.6	29,830	531.5	183
LF-5	11.8	7.8	7.910	0.377	69,373	1624.5	23,183	793.0	-	15.8	8.2	14.05	0.677	47,137	542.6	29,900	517.3	-
Ref.	^b^	[37]	[37]	^b^	^b^		^b^		[38,39,40,41,42,43]	^b^	[37]	[37]		^b^		^b^		[38,39,40,41,42,43]

^a^ Measurements are averages of three as indicated in the experimental section. Abbreviations: UF, Upper Fountain; MC, Monument Creek; LF, Lower Fountain. ^b^ Information provided in this work not previously published.
